# Female genital mutilation/cutting: sharing data and experiences to accelerate eradication and improve care

**DOI:** 10.1186/s12978-017-0361-y

**Published:** 2017-09-15

**Authors:** Jasmine Abdulcadir, Sophie Alexander, Elise Dubuc, Christina Pallitto, Patrick Petignat, Lale Say

**Affiliations:** 1 Department of Obstetrics and Gynecology, Geneva University Hospitals, Faculty of Medicine, University of Geneva, 30 Bld de la Cluse 1211, Geneva 14, Switzerland; 2 G3, Le réseau de la Francophonie, University of Geneva, Geneva, Switzerland; 3 G3, Le réseau de la Francophonie, Université Libre de Bruxelles, Brussels, Belgium; 4 G3, Le réseau de la Francophonie, University of Montreal, Montreal, Québec Canada; 5 Perinatal Unit and Reproductive health Unit (PERU), Ecole de Santé Publique, Université Libre de Bruxelles, Brussels, Belgium; 6 University of Montreal, Montreal, Québec Canada; 7 Department of Reproductive Health and Research, World Health Organization, Geneva, Switzerland

Female genital mutilation or cutting (FGM/C), as a topic, has evolved over the last eighty years, from being almost unheard of outside practicing countries [1], to a subject about which, there is now greater awareness. However, many misconceptions prevail. We support the idea that everyone needs to know basic facts about FGM/C, that all health care providers should be involved in avoiding new cases and trained to provide care for existing ones, and that beyond these consensual aspects, there are areas of doubt and lack of evidence which scientists and policy makers need to identify, understand and address. In this area of “expertise”, the present issue of RH contains abstracts from presentations and e-posters from a conference which took place in Geneva in March 2017 titled “Management and prevention of female genital mutilation/cutting: sharing data and experiences, improving collaboration”.

## What should everyone know about FGM/C?

Female Genital Mutilation or Cutting (FGM/C) involves the partial or total removal of the external female genitalia, or any other injury to the female genital organs for non-therapeutic reasons. It is mainly performed on children or adolescents and has an essentially ritual origin. There are no health benefits whatsoever, but severe complications can occur such a major bleeding and infection. FGM/C can also be responsible for negative long-term health outcomes, both somatic and mental [2]. The practice is prevalent in some countries in Africa, but also in parts of Asia and among some ethnic groups in South America. Worldwide 200 million women have undergone FGM/C, and more than three million girls are at risk every year [3]. Because of migration, over a million women with FGM/C are now living in high income countries where the health system needs to address this “new” condition. The World Health Organization (WHO) distinguishes four different types of FGM/C: type 1: the cutting of the prepuce or the glans of the clitoris; type 2: the excision of the labia with or without the cutting of the clitoris; type 3: the narrowing of the vaginal orifice suturing the labia with or without excision of the clitoris; and type 4: any other genital non-therapeutic alterations such as piercing, labial elongation and pricking [2]. There is evidence that FGM/C goes back at least to pharaonic times and that the practice is prevalent in Animists, Christians and Muslims. One of the first known formal opposition to the practice came from medical doctors in Egypt and from missionaries in Kenya, both in the early XX^th^ century [4]. In 1993, the United Nation General Assembly included FGM in resolution 48/104, the Declaration on the Elimination of Violence Against Women [5], and from 2003 sponsored the International Day of Zero Tolerance for Female Genital Mutilation, held every 6 February [6]. Most countries worldwide have legally banned the procedure. One of the present priorities is to accelerate the trend by adopting the most efficient and acceptable policies and achieve Sustainable Development Goal (SDG) 5, of gender equality, which includes the elimination of all forms of violence against women and girls by 2030 [7]. Such total elimination has occurred during the second half of the XX^th^ century for another traditional harmful practice, the millennial Chinese practice of foot binding, showing that such changes are possible. In addition to the primary goal of elimination, as long as FGM/C has not been totally stopped, there will be affected girls and women, for whom appropriate care must be provided.

## What should all professionals who are participants in eradication of new cases and better care of women affected know?

Many health care providers and other social service professionals provide care and services to women and girls with FGM/C, and need appropriate information and training on how to avoid new cases and provide better support and care to those who have already undergone the practice. These professionals are not only healthcare providers, but also psychologists and sexologists, social workers, asylum workers, advocates, interpreters, police staff, school nurses, and teachers, and probably many more.

Attitudes regarding FGM/C can be influenced by one’s personal views and the cultural context in which care is provided. Therefore, it is imperative that there are opportunities for providers to explore these influences and address their own attitudes to avoid bias and stigmatization around FGM/C, which could result in inappropriate care of girls and women who have already undergone FGM/C. In some areas, health care providers might also encounter families that request reinfibulation after delivery or a genital cutting, also as alternative less invasive forms, performed in a medical setting. Professionals working in this field need to have access to valid and evidence based guidance and tools for their work, including for example the WHO guidelines [8] and training materials [9], national guidelines where they exist, legal codes and policy statements, as well as information and education materials for women and their families, for example in schools.

## For policy makers, scientists and specialized care-givers; what are the research gaps, the implementation issues and the controversies; and do they matter?

### Research gaps

Research gaps in the health care context have been described [2, 10], and there is room for additional research to address these gaps on topics including timing of de-infibulation, the effectiveness of perineal physiotherapy, the best prevention strategies for health care providers to undertake, coping with sexual and psychological difficulties, clitoral reconstruction outcomes, and many more.

### Implementation issues in high prevalence and diaspora countries

There is some evidence that in many high prevalence countries the practice is decreasing, and also, that in countries with a high prevalence of type 3, infibulation is being replaced by less severe forms. However, the determinants of cessation of FGM/C remain elusive, and the process of abandonment varies from country to country. In diaspora countries as well, procedures vary extensively. Some have introduced mandatory reporting and recording. Others believe that such registration and reporting leads to stigma and that promoting trust with providers is more effective to bring about change.

### Controversies

Be it in high prevalence or diaspora countries, major controversies are present. For example, in some areas, health care providers perform FGM/C. This is known as medicalization, which is genital cutting by a healthcare provider in any setting and at any point in a woman’s life (2). Health-care providers increasingly carry out the practice mainly with the belief that health risks might be lower [11], and some stakeholders believe that medicalization is an intermediate and temporary step towards abandonment of FGM/C. However, WHO and more than ten other UN agencies and organizations released an interagency statement opposing medicalization of FGM/C [12].

Another debated topic is clitoral reconstruction, which in some diaspora settings is offered on a large scale without routine counseling and in others, in the context of careful counseling and psychosexual therapy that allow sometimes to meet the needs of women and go for surgery in few cases. Some health care providers consider clitoral reconstruction to be a right to regain something unjustly removed, while others are mainly focused on improving outcomes related to improved sexual function and reduced pain. While clitoral reconstruction can result in improved outcomes in some women, the safety and efficacy of the procedure is not yet known, and it is not clear whether and when the sexual counseling can also result in improved outcomes in the absence of surgery [13].

Another controversial topic relates to non-therapeutic genital surgery in adults. In general, in diaspora countries adult women who have undergone defibulation will be denied reinfibulation after giving birth, but the same women can access “genital cosmetic surgery”, which may even be covered through social security or insurance.

### How getting together and sharing data and experience can help to advance the field

At present, even though research on prevention, treatment of FGM/C and training of healthcare professionals seems to be increasingly implemented in high prevalence and diaspora countries, little data and experience are shared or compared across countries. The recent guidelines of WHO on the management of FGM/C, in fact underlined the need for more research to improve the evidence base; multicenter research on the subjects like defibulation, clitoral reconstruction or mental and sexual health after FGM/C, and of evidence-based healthcare professionals training, in particular for reducing FGM/C “medicalization”.

This supplement to Reproductive Health contains the abstracts of key-note lectures, accepted oral presentations and e-posters from the international experts’ meeting titled “Management and prevention of female genital mutilation/cutting: sharing data and experiences, improving collaboration”, which took place at the Department of Obstetrics and Gynaecology of the Geneva University Hospitals (Geneva, Switzerland) in March 13–14, 2017. The meeting was organized by the G3 de la Francophonie (a consortium of three French speaking universities: Geneva (UNIGE), Brussels (ULB) and Montreal (UM)), in collaboration with the Department of Reproductive Health and Research of the World Health Organization. Additional support and funding was received from the Swiss Network against Female Genital Mutilation/Cutting (FGM/C), and the Geneva University Hospitals. Participants came from four continents and 23 countries.

The goals of this supplement are to share data, research initiatives and experiences of healthcare professionals, researchers and other experts, from diaspora and FGM/C high prevalence countries. These abstracts are classified along the three main themes of the two day conference: i) Healthcare professionals’ training and curricula / medicalization of FGM/C; ii) Healthcare and prevention of FGM/C, and iii) Current evidence/consensus gaps. By definition these categories are somewhat arbitrary and overlap.

It is hoped that this supplement will allow dissemination of the very enriching exchanges that occurred and will encourage further networks and multicenter research to effectively prevent the continuation of female genital mutilation/cutting (FGM/C), improve health and understand the needs of women and girls who live with FGM/C.

### Next steps

Advocates, researchers and healthcare professionals have sometimes devoted a life time to preventing the procedure and treating complications of FGM/C, in both high prevalence and diaspora countries. Building on this momentum and experience can further propel this field forward. Collaboration and sharing data, thoughts, experiences and controversies can improve our work and research in eliminating myths and misconceptions; understanding how the eradication of FGM/C can be accelerated and improving the care and communication with girls and women with FGM/C and their partners.

*The ideas and conclusions reported in the abstracts presented in the supplement made by individual researchers and institutions are solely the responsibility of the authors and do not necessarily represent the views of the meeting’s organizers and their institutions.*
